# *Trifolium pratense* ‐Derived Exosome Improved Serum Biochemical Parameters and Pancreatic Genes in STZ‐Induced Diabetic Rats

**DOI:** 10.1002/edm2.70103

**Published:** 2025-08-31

**Authors:** Amir Hossein Khazaei, Azam Bozorgi, Elham Ghanbari, Maryam Bozorgi, Mozafar Khazaei

**Affiliations:** ^1^ Fertility and Infertility Research Center Health Technology Research Institute, Kermanshah University of Medical Sciences Kermanshah Iran; ^2^ Department of Tissue Engineering, School of Medicine Kermanshah University of Medical Sciences Kermanshah Iran; ^3^ Student Research Committee Kermanshah University of Medical Sciences Kermanshah Iran

**Keywords:** diabetes, plant‐derived exosomes, *Trifolium pratense*

## Abstract

**Introduction:**

Plant‐derived exosomes (PDEs) are promising nanotherapeutics for improving chronic diseases, such as diabetes mellitus. *Trifolium pratense* (*TP*) is a flowering herb with potent antioxidant and antidiabetic properties. The present study aimed to explore the diabetic‐healing effects of *TP*‐derived exosomes (*TPDEs*) in streptozotocin (STZ)‐induced diabetic rats.

**Methods:**

*TPDEs* were isolated using polyethylene glycol precipitation and serial centrifugation and characterised. STZ‐induced diabetic rats were treated with *TPDE* doses (0, 100, 200, and 400 μg/kg) for 28 days. Biochemical factors (fasting blood sugar (FBS), insulin, C‐peptide, total antioxidant capacity (TAC), and nitric oxide (NO)) were evaluated in serum samples. Also, the expression of *PDX1, insulin, NGN3*, and *SIRT1* genes in pancreas tissues was assessed using real‐time PCR.

**Results:**

*TPDE* treatment decreased the serum levels of FBS and NO while increasing c‐peptide, insulin, and TAC levels. It also significantly enhanced the expression of *insulin, PDX1, NGN3,* and *SIRT1* genes. *TPDEs* at doses of 100 to 200 μg/kg showed the most significant antidiabetic effects.

**Conclusion:**

TPDEs significantly improved diabetes‐induced alterations in serum insulin levels, antioxidant status, and pancreas‐related gene expression. It can be considered a novel complementary treatment for diabetes.

## Graphical abstract

*Trifolium pratense‐*derived exosomes improved the biochemical factors and pancreas regeneration in diabetic rats.

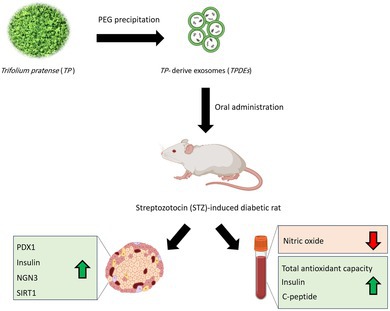

## 1 Introduction

Diabetes mellitus (DM) is a life‐threatening chronic disorder with increasing global prevalence; according to the International Diabetes Federation (IDF) report, approximately 536 million people had diabetes in 2021, anticipated to rise to 738 million by 2045 [1]. The main types of DM include type 1 DM (T1D), type 2 DM (T2D), and gestational DM (GDM), each with distinct pathophysiological states. T1D arises from an autoimmune disorder in which the immune system attacks and destroys insulin‐producing pancreatic β cells, resulting in absolute insulin deficiency [2]. T2D is a complex pathological condition characterised by inadequate insulin secretion and peripheral insulin resistance. GDM is a temporary hyperglycaemic condition observed during pregnancy due to hormonal changes and alterations in glucose homeostasis [3].

Oxidative stress (OS) plays a critical role in the pathogenesis and development of DM and related complications. Excessive reactive oxygen and nitrogen species exacerbate mitochondrial and endoplasmic reticulum stress, resulting in oxidative damage to β cells and ultimately leading to cell apoptosis. Furthermore, OS upregulates inflammatory signalling pathways, such as nuclear factor kappa B (NFκB), which give rise to T cell autoreactivity in T1D, hyperglycemia, and insulin resistance in T2D, as well as related outcomes like vascular dysfunction [4, 5].

Targeting OS using natural antioxidant compounds is a fascinating strategy to prevent cellular damage, reduce oxidative burden, and regulate glucose and lipid metabolism in diabetic patients [6]. Historically, medicinal and dietary plants have been used as food or traditional remedies to help reduce blood glucose levels and alleviate diabetes complications. Various medicinal herbs are used to treat DM via single or multiple mechanisms, including regulating glucose uptake, controlling insulin secretion, and reducing insulin resistance in target tissues [7].

Plant‐derived exosomes (PDEs) are a novel generation of bioactive compounds that have recently attracted much attention in biomedical applications. PDEs are biocompatible, biodegradable, and non‐toxic secretory vesicles in nanometre dimensions, with significant potential to cross biological barriers and deliver therapeutic elements to the target site [8]. PDEs reflect the structural and compositional nature of the parent cell from which they originate, carrying various bioactive molecules including DNA, proteins, lipids, mRNA, miRNA, and other metabolites [9]. PDEs exhibit outstanding anticancer [10], antioxidant and redox‐balancing [11], anti‐inflammatory [12], and tissue‐regenerative effects [13]. Exosomes derived from ginseng (GExos) restored diabetes‐induced endothelial dysfunction in full‐thickness diabetic skin wounds in mice by upregulating anaerobic glycolysis, downregulating OS, and stimulating angiogenesis and nascent vessel network reconstruction [8].

*Trifolium pratense* (*TP*), commonly known as red clover, is a perennial flowering plant belonging to the Leguminosae family, with a geographical distribution spanning Europe, Western Asia, and Northwest Africa [14]. *TP* is rich in phytochemicals, including flavonoids, isoflavones, phenolic glycosides, chalcones, and coumarins, as well as phytoestrogens [15]. The phytoconstituents in *TP* endow it with antioxidant, anti‐inflammatory, anticancer, neuroprotective, and cardioprotective effects, as well as beneficial influences on female reproductive health [16, 17]. Some investigations have explored the impact of *TP* extracts on relieving the progression of diabetes and its subsequent complications. A study showed that extracts from *TP* flowering tops decreased hyperglycaemia and insulin resistance in T2D rats, associated with increased expression of sirtuin 1 (*SIRT1*) in pancreatic tissue [18]. Moreover, *TP* diminished diabetes‐mediated testicular damage in diabetic male rats by enhancing antioxidant capacity and restoring sperm viability and motility [19].

In the present study, we aimed to evaluate the effect of *TP*‐derived exosomes (*TPDEs*) on the biochemical parameters of hyperglycaemia and pancreatic gene expression in an STZ‐induced diabetic model in Wistar rats.

## 2 Materials and Methods

### 2.1 *TPDE* Isolation and Characterisation

Fresh *TP* plant was prepared from the herbal farm of the School of Pharmacy, Kermanshah University of Medical Sciences, Kermanshah, Iran. *TP* parts were cleaned and washed with phosphate‐buffered saline (PBS) and then chopped into small pieces. Then, *TP* fragments were incubated in PBS overnight under gentle shaking at room temperature (RT). Next, the extract was subjected to a two‐step centrifugation at 4000 rpm for 20 min and 10,000 rpm for 40 min to isolate large and fine particles. The obtained supernatant was filtered through a 0.22 μm filter, and exosomes were isolated using a Polyethylene glycol precipitation method (12% PEG 8000, Cat no: 25322‐68‐3, Biovest) overnight, followed by centrifugation at 8000 rpm for 30 min [20]. The exosome pellet was collected, resuspended in PBS, and stored at −20°C until further use. The protein content of *TPDE* was determined using a BCA kit (KBCA96, Kiazist, Iran) according to the manufacturer's instructions. The size distribution and zeta potential were evaluated using a DLS/zeta sizer machine (Malvern Instrument, UK). The *TPDE* pellet was resuspended and diluted (1:100) in PBS and then measured at 25°C with a scattering angle of 90° [21].

### 2.2 In Vivo Experiment and *TPDE* Dose Determination

25 male Wistar rats (6–8 weeks, 190 ± 10 g weight) were divided into five groups (*n* = 5): control, untreated diabetic, and diabetic groups treated with varying doses of *TPDEs*. Diabetes was induced in fasting rats by intraperitoneal (i.p) injection of a single dose of STZ (60 mg/kg, dissolved in citrate buffer) [22]. Diabetes induction was confirmed by measuring fasting blood sugar (FBS) on day 5 post‐STZ injection by FBS value > 250 mg/dL. The *TPDE* dose, treatment duration, and route of administration were selected based on similar reports described in the previous literature [23]. Therefore, three doses of *TPDE* (100, 200, and 400 μg/kg of body weight) were administered intraperitoneally daily for 28 days to diabetic rats.

Animals were weighed on day 28, and FBS was measured using a glucometer (Bionem, Taichung City, Taiwan). After the 28‐day treatment, the animals were euthanised, and blood samples were collected and centrifuged. The sera were then separated and stored at −20°C for subsequent biochemical measurements. Moreover, the fresh pancreatic tissues were immediately dissected and placed in liquid nitrogen for gene expression analysis.

### 2.3 Measurement of Serum Biochemical Markers

#### 2.3.1 Nitric Oxide (NO) Measurement

NO levels were assessed by measuring nitrite (NO₂^−^) and nitrate (NO₃^−^) using the colorimetric Griess assay, as described previously [24]. Briefly, serum samples were deproteinised using zinc sulfate and centrifugation. Then, the deproteinised samples were mixed with ethylene diamine dihydrochloride (0.1%) and sulfanilamide (2%), followed by incubation at 37°C for 30 min. The sample optical density (OD) values were read at 540 and 630 nm wavelengths using a microplate ELISA reader. NO values were calculated against the sodium nitrate standard serial solution (0 to 200 μM).

#### 2.3.2 Total Antioxidant Capacity (TAC) Measurement

Serum TAC levels were measured using a colorimetric FRAP method based on the reduction of Fe^3+^ to Fe^2+^ in the presence of tripyridyltriazine, and the sample ODs were read at a wavelength of 593 nm using a microplate ELISA reader (STAT FAX2100, USA) [25].

#### 2.3.3 Insulin and C‐Peptide Measurement

Serum levels of insulin and C‐peptide were measured using specific ELISA kits for rats (insulin kit Cat no EL0023Ra, C‐peptide Cat no SL0200Ra, SunLong Biotech, China). For insulin assessment, 20 μL of serum samples were mixed with 80 μL of assay buffer and 50 μL of insulin conjugate, followed by incubation at 25°C for 90 min. After aspirating and washing, 100 μL of Streptavidin‐HRP was added to sample wells and incubated at 25°C for 30 min. After the solution was aspirated, 100 μL of substrate solution was added to each well and incubated at 25°C for 5 min in the dark. Finally, 100 μL of stop solution was added to wells, and the sample OD was read at 450 nm.

For C‐peptide assessment, the sample and dilution buffer were mixed in each well at a 1:4 ratio, incubated at 37°C for 30 min, and then the wells were washed. Then, 50 μL HRP‐conjugate reagent was added to each well, followed by incubation and washing, as described earlier. Next, equal volumes of Chromogen Solutions A and B were added to the wells, and the plate was incubated at 37°C for 15 min in the dark. Finally, 50 μL of Stop Solution was added to the wells, and the sample OD was read at 450 nm. Insulin and C‐peptide values were computed against relative serial dilution standard curves.

### 2.4 Real‐Time Polymerase Chain Reaction (PCR)

The expression of pancreatic‐related genes, including *Insulin, pancreatic duodenal homeobox 1* (*PDX1*), *neurogenin‐3* (*NGN3*), and *SIRT1*, was evaluated using the Real‐time PCR method. Fresh pancreas tissues were collected, homogenised, and subjected to RNA extraction using the TRIzol reagent (Life Biolab, Hanseatic, Hamburg, Germany). After determining the RNA concentration using the NanoDrop spectrophotometer (Thermo Scientific), 1 μg of extracted RNA was used for cDNA synthesis (Thermo Scientific RevertAid First Strand cDNA synthesis kit, UK). Real‐time PCR was carried out using cDNA, specific Forward and Reverse primers, and Amplicon master mix (AMPLICON, Stenhuggervej, Denmark) in the Step One Real‐time machine (Applied Biosystems, USA), where the amplification program consisted of 15 min at 95°C, followed by 40 cycles of denaturation (95°C, 15 s), annealing and extension (60°C, 60 s). Relative gene expression was reported as the Fold change = 2^(−ΔΔCT)^, where the values were normalised against the Glyceraldehyde‐3‐phosphate dehydrogenase (*GAPDH*) as the internal reference gene [26]. Forward and Reverse primers are listed in Table 1.

**TABLE 1:** Forward (F) and Reverse (R) primers used for Real‐time PCR.

Gene	Gene ID	Sequence (5′‐3′)	Length	Annealing temp.
Rat‐PDX1	3651	F: CCCGAGCTTCTGAAAACTTTG R: CTTTTCATTGTCCTCAGTTGGG	21 22	60 60
Rat‐ Insulin	24506	F: CCATCAGCAAGCAGGTCAT R: TGTGTAGAAGAAACCACGTTCC	19 22	60 60
Rat‐ NGN‐3	60329	F: TCCAGACGCAATTTACTCCAG R: CTAGTTCTCCGGGCTCAAAAG	21 21	60 60
Rat‐ SIRT1	309757	F: CCAGATTTCAAGGCTGTTGGTTCC R: CCACAGGAACTAGAGGATAAGGCGT	24 25	60 60
Rat‐ GAPDH	24383	F: TGGAGTCTACTGGCGTCTT R: TGTCATATTTCTCGTGGTTCA	19 21	60 60

### 2.5 Data Analysis

The normality of the data distribution was analysed using the Kolmogorov–Smirnov test. Quantitative data were analysed using GraphPad Prism software version 7 (GraphPad Software, San Diego, CA, USA). The results were compared among groups using one‐way analysis of variance (ANOVA) and Tukey post hoc tests at a significance level of *p* < 0.05.

## 3 Results

### 3.1 TPDE Isolation and Characterisation

**FIGURE 1:**
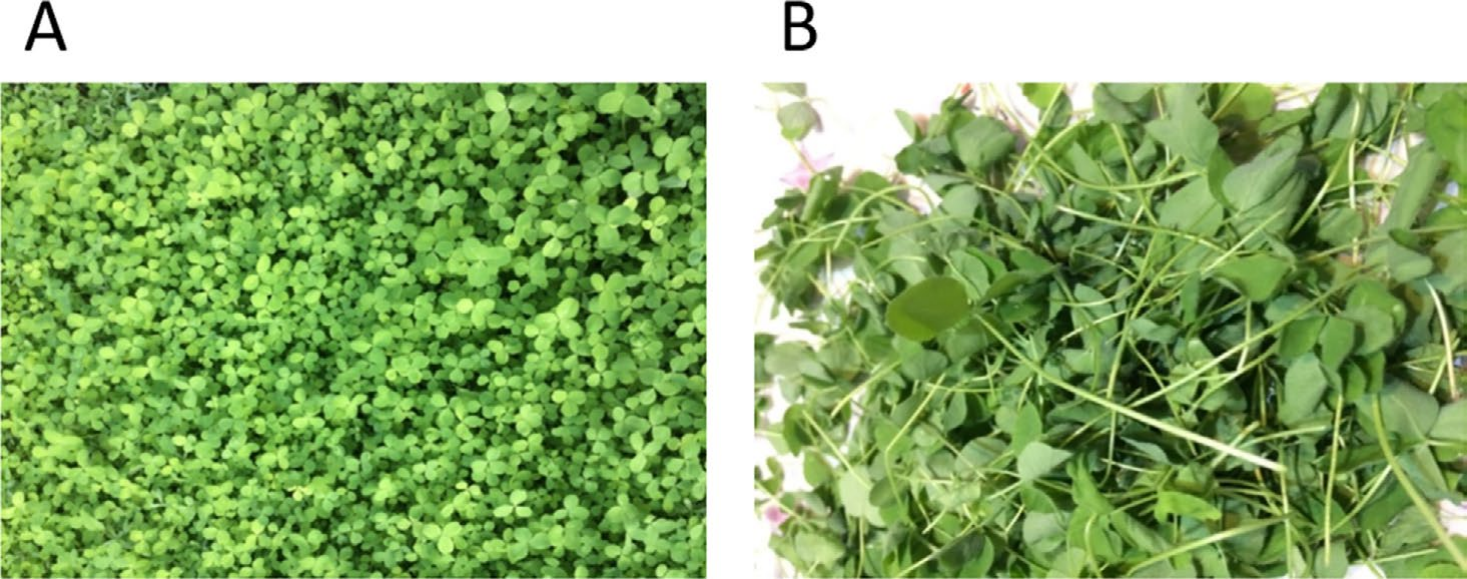
*T. pratense* fresh plant. (A) Before harvesting; (B) Cleaned aerial parts.

**FIGURE 2:**
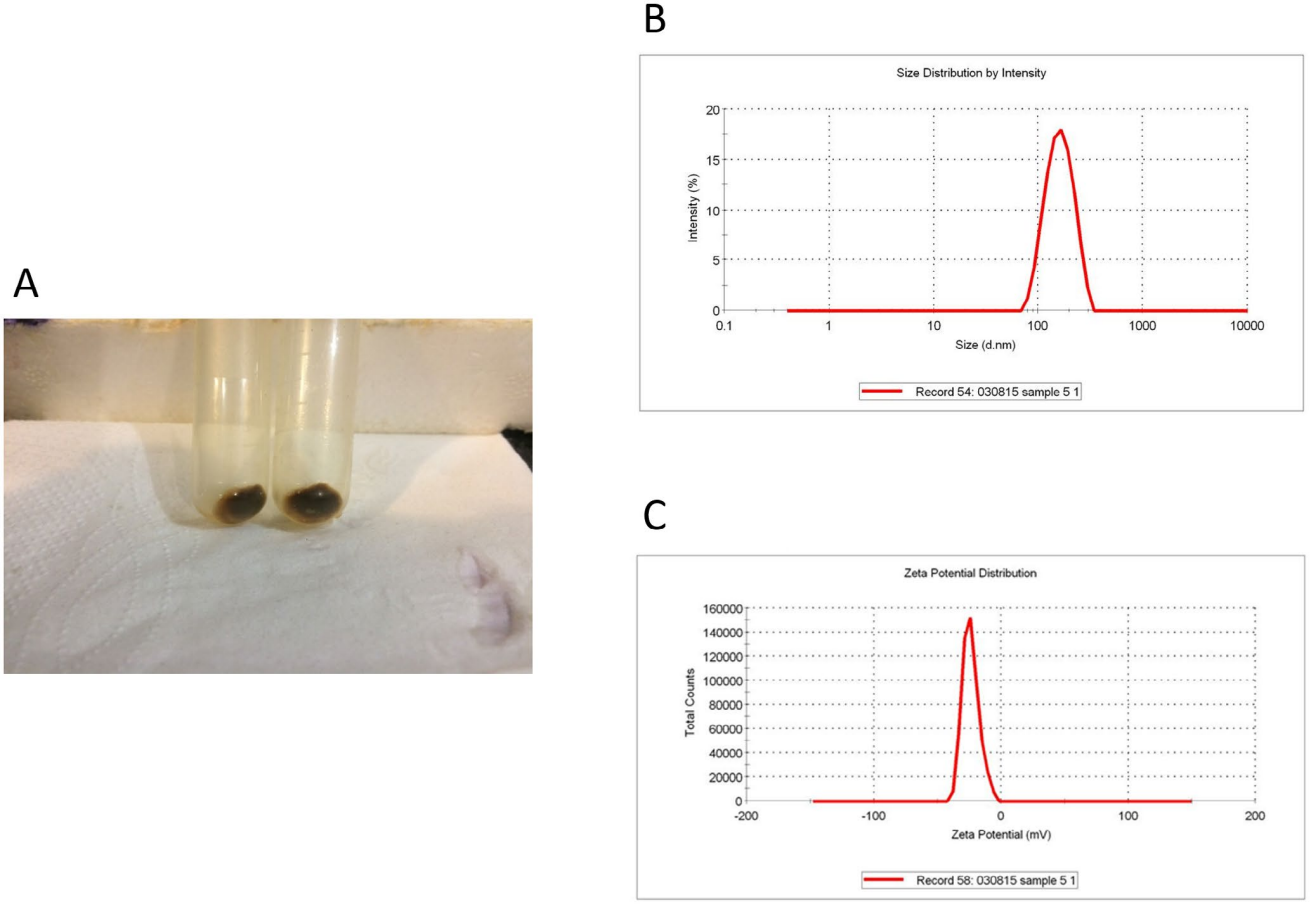
Characterisation of *TPDE*. (A) *TPDEs* participated using PEG; (B) DLS data; (C) Zeta potential data. DLS, dynamic light scattering; PEG, polyethylene glycol; *TPDE*, *T. pratense* ‐derived exosome.

Fresh *TP* was harvested (Figure 1A,B), and *TPDEs* were extracted using PEG‐based precipitation and centrifugation (Figure 2A). The size (mean diameter), zeta potential, and polydispersity index (PDI) of *TPDEs* were 167.3 nm, −23.9 mV, and 0.33 (Figure 2B,C).

### 3.2 FBS and Body Weight

**FIGURE 3:**
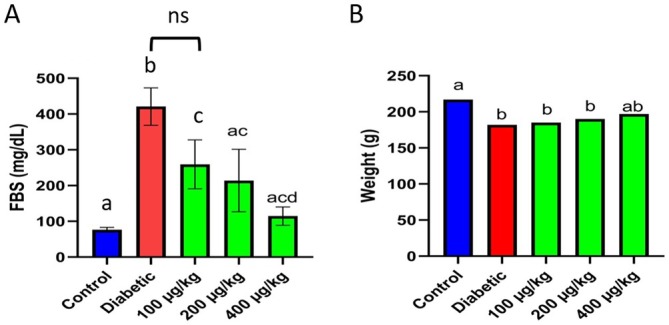
The analysis of FBS and body weight in healthy (control) and STZ‐induced diabetic rats treated with *TPDEs*. (A) FBS; (B) Body weight. Different letters indicate significant differences between groups. B, body weight; FBS, fasting blood sugar. (a, b: *p* < 0.001, c, d: *p* < 0.05, ac: no significant differences with a and c, ns: non‐significant).

The FBS values and animal weights were measured on day 28 of the experiment (Figure 3). The results showed that FBS levels increased significantly in the diabetic group compared to the healthy control treatment groups (a, b: *p* < 0.001). *TPDE* treatment reduced FBS levels remarkably in a dose‐dependent manner, where the FBS level in rats that received *TPDE* at 400 μg/kg dropped to that of the control group (c, d: *p* < 0.05, ac: no significant differences with a and c, ns: non‐significant) (Figure 3A). Furthermore, body weight was reduced in diabetic rats compared with the healthy control (a, b: *p* < 0.05), and *TPDE* treatment improved body weight dose‐dependently (b: *p* < 0.05, ab: no significant differences with a and b) (Figure 3B).

### 3.3 NO and TAC Assessment

**FIGURE 4:**
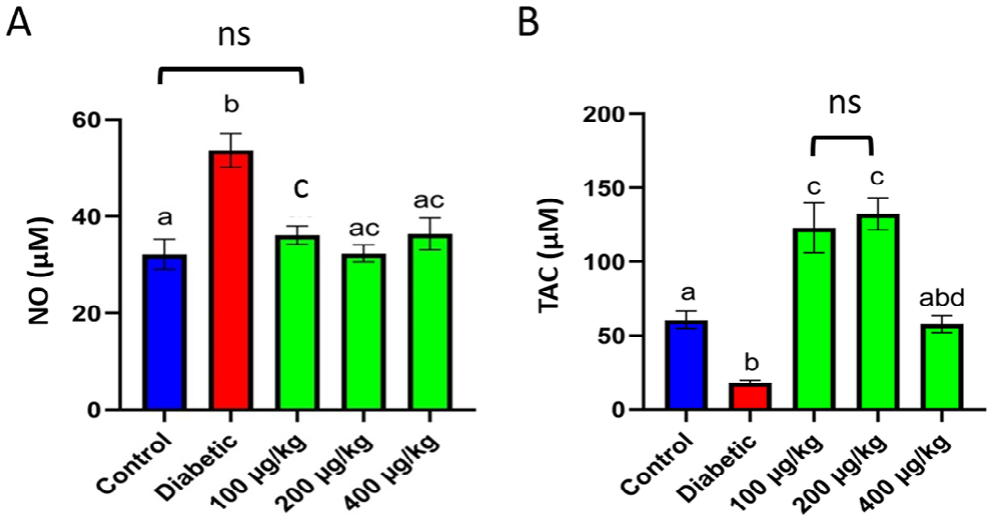
The analysis of NO and TAC in healthy (control) and STZ‐induced diabetic rats treated with *TPDEs*. (A) NO; (B) TAC. Different letters indicate significant differences between groups. NO, nitric oxide; TAC, total antioxidant capacity (a, b: *p* < 0.05, c, d: *p* < 0.001, ab: no significant differences with a and b, ns: non‐significant).

The results from the Griess assay showed that NO levels were significantly higher in diabetic rats than in the control group (a, b: *p* < 0.05). In contrast, NO levels reduced notably in treated groups (c: *p* < 0.001), but no differences were observed between the treatment groups and the control group (ac: no significant differences with a and c) (Figure 4A). TAC levels were diminished in the diabetic rats compared to the control group (a, b: *p* < 0.001). TAC was improved in *TPDE* groups with significant increments in 100 and 200 μg/kg groups (c, d: *p* < 0.001, ab: no significant differences with a and b, ns: non‐significant) (Figure 4B).

### 3.4 Insulin and C‐Peptide Assessment

**FIGURE 5:**
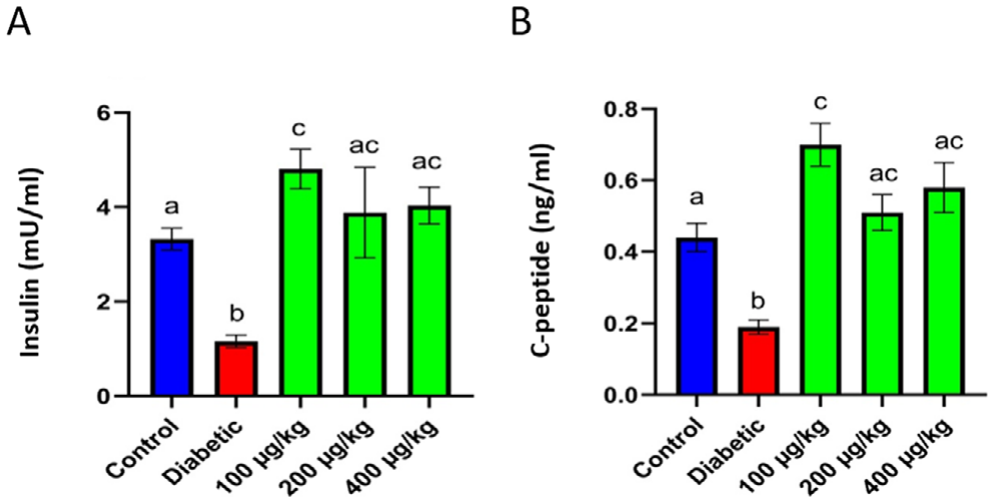
The analysis of insulin and C‐C‐peptide in healthy (control) and STZ‐induced diabetic rats treated with *TPDEs*. (A) Insulin; (B) C‐C‐peptide. Different letters indicate significant differences between groups (a, b, c: *p* < 0.001, ac: no significant differences with a and c).

The serum analysis data manifested that insulin and C‐peptide values were remarkably lower in the diabetic group than in the control group (a, b: *p* < 0.001). Moreover, insulin and C‐peptide levels were elevated in diabetic rats treated with TPDEs, with the most significant increase observed in animals treated with a 100 μg/kg dose (c: *p* < 0.001, ac: no significant differences with a and c) (Figure 5A,B).

### 3.5 Gene Expression Assessment

**FIGURE 6:**
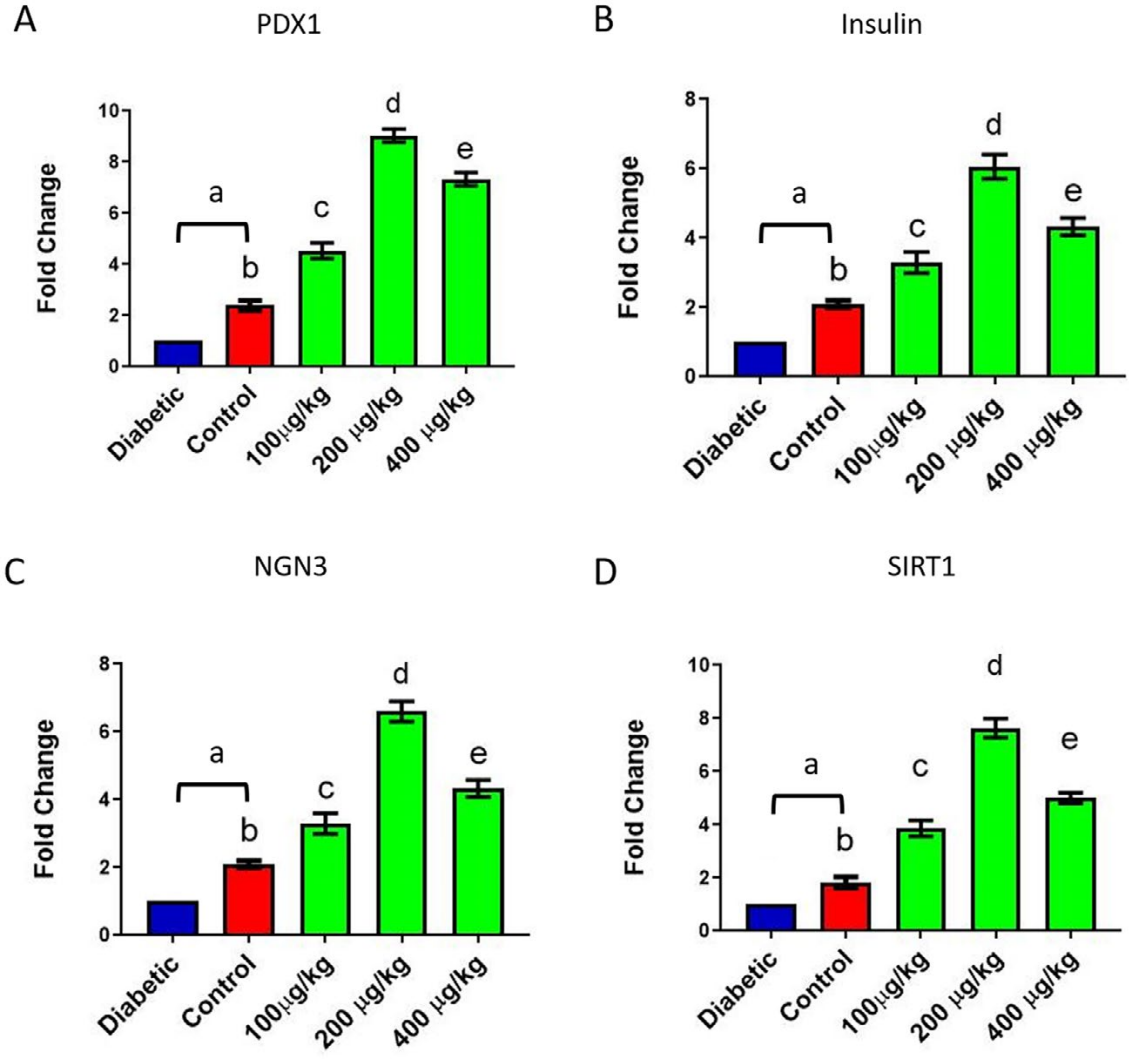
The expression of *PDX1*, *insulin*, *NGN3*, and *SIRT1* in pancreas tissue of healthy (control) and STZ‐induced diabetic rats treated with *TPDEs*. (A) *PDX1*, (B) *Insulin*, (C) *NGN3*; (D) *SIRT1*. *NGN3*, Neurogenin 3; *PDX1*, pancreatic and duodenal homeobox 1; *SIRT1*, Sirtuin 1. (a: *p* < 0.05, b–e: *p* < 0.001).

The expression of *insulin, PDX1, NGN3, and SIRT1* in pancreas tissues was evaluated on day 28 of the experiment. *PDX1* expression was significantly reduced in the diabetic group compared to the healthy control group (a: *p* < 0.05). *PDX1* was overexpressed in the treatment groups, with a notable difference from the diabetic and healthy groups (b, c, d, e: *p* < 0.001) (Figure 6A). *Insulin* levels in diabetic rats were significantly lower than those in the healthy group (a: *p* < 0.05). TPDE treatment caused an increment in *insulin* expression with the highest value in the 200 μg/kg groups (b, c, d, e: *p* < 0.001) (Figure 6B). NGN3 decreased prominently in diabetic rats compared to the healthy group (a: *p* < 0.05). In contrast, diabetic rats that received *TPDE*s experienced much higher levels of NGN3 than diabetic and healthy rats, with the highest value in 200 μg/kg doses (b, c, d, e: *p* < 0.001) (Figure 6C). SIRT1 notably diminished in the diabetic rats compared to the control group (a: *p* < 0.05). *TPDE*‐treated groups exhibited elevated *SIRT1* expression, and the most significant alteration was observed in the 200 μg/kg groups (b, c, d, e: *p* < 0.001) (Figure 6D).

## 4 Discussion

In the present study, the effect of *TPDEs* on improving serum biochemical and antioxidant parameters, as well as the expression of pancreatic‐related genes, was evaluated in the STZ‐induced diabetic rat model. The results showed that *TPDEs* improved diabetic parameters, including FBS, insulin, C‐peptide, and antioxidant parameters (NO, TAC) in serum and enhanced pancreatic gene expression (*insulin, PDX1, NGN3*, and *SIRT1*). Our previous in vitro study demonstrated that the *TP* hydroalcoholic extract exhibited protective effects against STZ‐induced toxicity and apoptosis in diabetic RIN‐5F cells, significantly increasing the insulin concentration in the culture medium [27]. Moreover, our in vivo investigation revealed that the TP extract had similar effects on reducing hyperglycemia and NO levels, as well as ameliorating TAC, in STZ‐induced diabetic rats. *TP* extract also restored the structural abnormalities of testicular tissue and improved sperm quality [19].

OS caused by the imbalance between free radical generation and scavenging plays a crucial role in the pathophysiology and development of T1D and T2D, as well as relevant complications [28, 29]. OS prompts mitochondrial and endoplasmic reticulum stress, leading to organelle dysfunction and protein uncoupling, which contribute to glucose intolerance, impaired insulin signalling, and apoptotic cell injury [30]. Natural plant extracts and their active constituents, with potent antioxidant properties, are beneficial in reducing hyperglycaemia, enhancing insulin signalling and resistance, and promoting β cell regeneration and function [31, 32]. PDEs are plant‐extracted, membrane‐bound nanostructures with excellent biocompatibility and lower toxicity and immunogenicity compared to synthetic and mammalian cell‐derived nanoparticles, making them an ideal option for treating diseases and delivering therapeutic compounds [33].

TP's potent antioxidant and antidiabetic properties are attributed to its high content of polysaccharides. Zhang et al. demonstrated that TP contained high amounts of polysaccharides, including glucose, galacturonic acid, arabinose, and galactose, with potent ROS scavenging and hypoglycaemic activities [34]. Bajaj et al. demonstrated that miRNAs in Ginger‐derived exosome‐like nanoparticles (G‐ELNs) decreased FBS levels and improved glucose tolerance in diabetic mice by modulating OS and phosphatidylinositol 3‐kinase (PI3K)/Akt‐2 pathways [35].

Pancreas regeneration and β cell replacement therapy outperform insulin therapy during DM, especially in T1D cases that experience immune‐mediated β cell destruction and loss of function. Thus, therapeutic strategies to enhance β cell proliferation and differentiation while preventing apoptosis are crucial in disease management and treatment [36, 37]. In the present work, treating diabetic rats with *TPDEs* significantly refined the expression of pancreas‐specific genes *insulin, PDX1, NGN3*, and *SIRT1*. *PDX1* is the main transcription factor essential for the formation of early pancreatic progenitor and β cell function, and it is indicative of pancreas regeneration following injury [38, 39]. A recent study demonstrated that *PDX1* administration enhanced pancreas and β cell regeneration in STZ‐induced diabetic animals [40]. Increased *insulin* expression confirmed that *TPDE* treatment successfully restored β cell population and function in diabetic rats. *NGN3* is a transcription factor critical for endocrine lineage specification and differentiation [39]. Casteele et al. indicated that *NGN3* contributed to β‐cell neogenesis and proliferation in injured adult mice [41].

*SIRT1* is necessary for pancreas development and β cell generation by deacetylating *FOXA2* on the *PDX1* promoter and regulating its transcription [42]. A study showed that *SIRT1* promoted β‐cell regeneration, along with NGN3 overexpression, which is regulated by AMP‐activated protein kinase (AMPK) signalling‐mediated fatty acid oxidation (FAO) [43]. Resveratrol‐activated *SIRT1* overexpression protected RIN‐m5F pancreatic β cells against IL‐1β and IFN‐γ‐induced damage by suppressing Nuclear Factor‐κB (NF‐κB) signalling pathway [44]. Transplanting adipose tissue‐derived stem cells pre‐incubated with Epigallocatechin gallate (EGCG) in STZ‐induced T1D rats increased pancreas regeneration by upregulating the *SIRT1* signalling pathway and modulating OS [45]. In conclusion, *TPDE* showed great potential to be considered a novel therapeutic option for treating DM. However, more in vitro and in vivo investigations are needed to elucidate their precise mechanisms of action and therapeutic prospects.

## Author Contributions

**Amir Hossein Khazaei:** validation, visualization, writing – original draft preparation. **Azam Bozorgi:** visualization, writing – original draft preparation. **Elham Ghanbari:** formal analysis, investigation. **Maryam Bozorgi:** formal analysis, investigation. **Mozafar Khazaei:** conceptualization, funding acquisition, investigation. All authors participated in writing – review and editing the manuscript.

## Ethics Statement

This experiment was conducted in accordance with the guidelines of the Ethics Committee of Kermanshah University of Medical Sciences [Ethical code: IR.KUMS.AEC.1402.036].

## Conflicts of Interest

The authors declare no conflicts of interest.

## Data Availability

The data that support the findings of this study are available from the corresponding author upon reasonable request.
